# Is AI overhyped?

**DOI:** 10.1016/j.patter.2025.101418

**Published:** 2025-11-14

**Authors:** Jason H. Moore, Anand K. Gavai, Yingji Xia, Mohammadamin Mahmanzar, Youping Deng

## Abstract

In this People of Data, we asked five researchers, including three members of the journal’s advisory board, whether they feel AI technologies are currently overhyped. Their responses reveal both optimism about the future impact of these technologies and serious concerns about overblown expectations and uncritical applications.

## Main text

**Figure:**
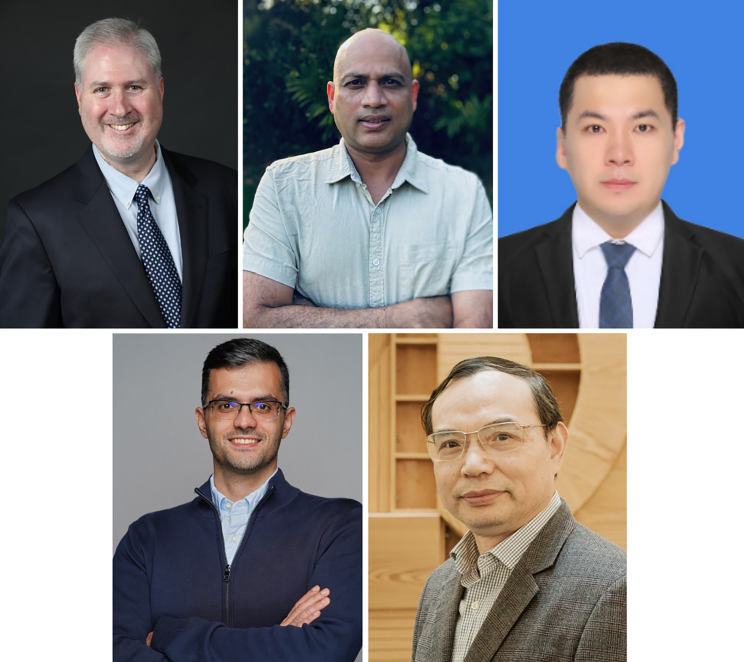
Clockwise from top left: Jason H. Moore, Anand K. Gavai, Yingji Xia, Mohammadamin Mahmanzar, and Youping Deng.

### Jason H. Moore

It has long been recognized that data engineering consumes much of the time that data scientists spend on the analysis of big data. This time is often spent on labor-intensive tasks such as cleaning and standardizing datasets, performing rigorous quality control, merging heterogeneous data from disparate sources, detecting and mitigating measurement error and bias, and conducting analyses that lead to reproducible models and generalizable predictions. The holy grail of data science is to automate data processing and management to free up time to focus on model building, evaluation, interpretation, explainability, deployment, and auditing. Recent developments in large language models (LLMs), deep learning, and high-performance computing have given rise to agentic artificial intelligence (AI) methods with autonomous agents that can break workflows into modular tasks that specialized AI agents can execute quickly and accurately under the oversight of supervisory “manager” and “evaluation” agents. If realized, this approach could dramatically accelerate data processing and analysis, save valuable financial resources, and shift the focus of researchers and analysts away from tedious data preparation toward creativity, discovery, and translation.

Despite this promise, skepticism remains over whether agentic AI can perform these tasks as effectively as humans. Real-world data are inherently complex, dynamic, and context-dependent, which makes it difficult for AI systems trained on fixed benchmarks to generalize effectively. This is particularly true in biomedical research, where every data element may be subject to time-dependent drift, varying sources of noise, or unique measurement error that requires expert interpretation and guidance that can only come from the humans who understand the instruments and assays generating the data. Many widely reported AI breakthroughs emphasize performance on static metrics or curated benchmark datasets that fail to capture the unpredictable and evolving nature of applied settings, perpetuating a cycle of hype that often outpaces capability. Further, LLMs and deep learning systems, which power many AI agents, are often opaque and can propagate or amplify errors through cascading feedback loops. This creates significant accountability and debugging challenges. These factors underscore the importance of maintaining humans in the loop, prioritizing reproducibility and explainability, and ensuring ethical and regulatory oversight as these systems mature. While AI hype has historically driven innovation, from expert systems in the 1980s to today’s generative AI, it is reality, grounded in careful domain expertise and rigorous validation, that tempers expectations and reminds us that human insight and hard work remain indispensable.

### Anand K. Gavai

The question arises from a widening gap between ambitious claims and reproducible outcomes, exacerbated by the influx of individuals engaging with AI without foundational skills in data or programming. In my research with farm data across Europe, AI systems intended to predict food safety risks were published as polished reports but remained untested beyond initial demonstrations. This gap undermines confidence among stakeholders and fuels skepticism about AI’s transformative claims.

Academic incentives reward novelty over validation, attracting those who discuss algorithms without coding, data without analysis, or AI without model construction, often securing funding through rhetoric rather than reproducible results. I have seen projects hailed as innovative that, behind the scenes, relied on trivial methodologies or collapsed when confronted with heterogeneous conditions. These experiences reflect a reproducibility crisis, with fewer than 30% of AI papers providing accessible code.^1^ While excluding nontechnical contributors risks gatekeeping, their unchecked involvement without rigor can mislead policy and misallocate resources. The challenge is how to broaden participation without diluting accountability.

Yet dismissing AI as hype ignores its real contributions. Validated systems in radiology and logistics already deliver measurable benefits. In my work on AI-driven nutrition, retrieval-augmented models grounded in dietary guidelines have supported public health, demonstrating AI’s potential when rigorously validated.^2^ These consistent but modest successes contrast with the inflated claims that dominate headlines.

Industry reports argue that hype stimulates investment. But this influx often directs resources to fragile systems while neglecting robustness or bias mitigation. The ethical stakes are high: flawed algorithms have misallocated healthcare to disadvantaged groups, and unmet promises from autonomous vehicles to “smart” agriculture have eroded public trust.^3^ These failures highlight two cultures of AI: one performative, optimized for visibility, and another operational, focused on deployment. Shifting toward the latter faces resistance from academic publication pressures and industry profit motives, compounded by literacy gaps that equate rhetoric with expertise.

Responsible innovation offers a path forward. Mandating code sharing, tying funding to deployment outcomes, and fostering genuine interdisciplinary collaboration can strengthen accountability. Yet reforms also risk reinforcing technical elitism or oversimplifying ethical complexities, underscoring the difficulty of systemic change.

So, is AI overhyped? Yes, when speculative narratives outpace validated outcomes, driven by rhetoric over rigor. But it is also underappreciated where reproducible, less glamorous systems already work. The challenge is to redirect AI’s trajectory from spectacle to accountability so that it earns trust as science, not performance.

### Yingji Xia

The answer to this question heavily depends on context. From a long-term technology advancement perspective, the trial-and-error mode is inevitable for AI to ultimately achieve its maturity. All endeavors are regarded as worthy and beneficial.

However, the (capital) markets might oversell the technology to common people to make more profits. For instance, skyrocketing attention has been drawn to LLMs, such as various chatbots, which are commonly reachable and enjoyable worldwide. While academics are still exploring the LLMs’ application boundaries and reliabilities due to heavy intrinsic hallucinations, the market size has already grown to about $5.73 billion in 2024 and is projected to be $7.79 billion in 2025.^4^ I suppose the subsequent impacts may be controversial.

Another (very personal) concern is whether the current trend of AI development will hurt mechanism-level knowledge discovery research. In my areas of expertise, namely autonomous driving and computer vision studies, there are fewer of these “hardcore/old-school” research papers in recent years. As new emerging architectures of neural networks can almost “fit everything,” I worry that younger researchers will never care about the “whys” and “hows” behind the scientific phenomena and instead focus only on benchmarks and accuracy measurements.

I also agree with Dr. Moore’s comment on the biased benchmarking issue. Many intrinsic factors in datasets, other than simple signal drift or colored noise, can negatively impact so-called “standardized benchmarks.” For instance, autonomous driving studies may divide a specific vehicle trajectory dataset collected in a fixed yet small spatiotemporal range into training and testing sets and then train models to mimic the specific trajectory generation manners in the given dataset. After obtaining high accuracy or equivalent measures, researchers may claim that their models can drive like humans. However, human driving behaviors are highly heterogeneous and irrational (which is the fact for most human-involved tasks). That is, you cannot claim that driving behaviors in United States and Germany are identical, nor can you do so for those of the 1980s, 2000s, and 2025. Driving intentions and behaviors on highways, urban roads, and rural roads are also unique, and other conditions like weather, light, or humidity only add more complexity. All of these diverse data characteristics and uncertainties can weaken the theoretical and practical contributions of subsequent studies and form stubborn bottlenecks for AI-driven research. Over 99% model accuracy, or something similar, is definitely not equal to high performance in real tasks and does not guarantee model credibility or generalizability.

### Mohammadamin Mahmanzar and Youping Deng

AI is changing many aspects of our daily lives, from healthcare and finance to entertainment and education. However, the excitement around AI often exaggerates what it can realistically do today or even in the near future. The question isn’t whether AI is useful—it clearly is—but whether expectations around AI have become too high, creating misunderstandings about its nature.

AI has demonstrated remarkable capabilities, such as recognizing patterns and images; diagnosing medical conditions; performing natural language processing, including tasks such as language understanding; predicting trends and outcomes; and automating defined tasks. For example, chatbots are able to write like humans, and in healthcare, AI helps spot diseases such as eye conditions better than common diagnostic methods.^5^

The excitement around AI and the media’s frequent tendency to present it in a sensational way suggests that AI will soon take over jobs, society, or even humanity, which can cause concern and unrealistic excitement. Movies and ads make it seem like AI can fix everything, from saving the planet to running a whole company. But is that feasible or not true yet? AI is great at specific information-dependent jobs but has issues with scenarios where it must think broadly like a human, limited energy resources, and reliance on accurate data. So, at this date, AI is still greatly dependent on human oversight, especially in critical, sensitive, and decision-making areas.

AI responses are generated based on data, information, and the AI’s ability to recognize the patterns and relationships within data, allowing the AI to produce more logical outputs. So, AI is currently struggling with tasks requiring it to demonstrate human empathy, understand context, and make moral judgments. For example, AI-powered chatbots can seem humanlike in conversation, but they often have difficulties with more complex or emotional interactions. While popular chatbots like ChatGPT or Claude communicate impressively, they can’t yet truly represent human creativity, emotional depth, or original ideas.

The hype about AI could originate from financial incentives. Companies call simple programs “AI-powered” or “AI-driven” to signal the advanced performance of applications to attract investors or customers. The problem could arise when service providers make unrealistic promises about their products or services to encourage early adoption without carefully considering the ethical, social, and legal implications. This kind of exaggeration can lead to disappointment when technology doesn’t live up to expectations.

Still, saying AI is all hype is neither accurate nor fair. AI’s products are helping to facilitate our daily life, much like the invention of the wheel once transformed human mobility. Early societies relied on limited physical and animal power until the wheel improved mobility. The combination of wheel and carriage design allowed both faster and heavier load movement. The invention of the steam engine was another revolution that tried to overcome previous limitations. In the same way, as computers have solved problems ranging from simple to complex over different periods, their architecture has also evolved and moved from simple structures and algorithms to complex networks and models. This progress has made life easier for people while also creating new needs and raising expectations. Going forward, jobs will disappear and new careers will emerge as technologies are invented to address human needs and overcome previous limitations, thereby also opening doors to new expectations. Hence, to maintain public trust in AI, it’s necessary that scientists, technologists, policymakers, and journalists talk openly and accurately about AI’s real abilities and limitations. Every claim about AI should be paired with its boundaries, just as every infrastructure project comes with safety standards.

AI is still at the beginning of its journey and must overcome significant challenges such as outdated knowledge, lack of reliable source attribution, and difficulty with real-time updates. In addition, problems like bias, transparency, and energy consumption remain unsolved. These are not signs of failure, but they are natural stages in technological evolution.

AI will not replace us, but it will serve us best when treated as a tool whose success depends on how wisely we use it and how well we align it with human values.

## Acknowledgments

Y.X. acknowledges support from the National Natural Science Foundation of China under grant 72401256. A.K.G. acknowledges support through the “High Tech for a Sustainable Future” capacity building program of the 4TU.Federation in the Netherlands.

## Declaration of interests

Y.D., Y.X., and J.H.M. serve as advisory board members for *Patterns*.
